# Molecular analysis of the choroideremia gene related clinical findings in two families with choroideremia

**Published:** 2011-09-30

**Authors:** Ying Lin, Xialin Liu, Lixia Luo, Bo Qu, Shuhong Jiang, Huiqin Yang, Xuanwei Liang, Shaobi Ye, Yizhi Liu

**Affiliations:** 1 State Key Laboratory of Ophthalmology, Zhongshan Ophthalmic Center, Sun Yat-sen University, Guangzhou, China; 2 People’s Hospital of Inner Mongolia, Hohhot, China

## Abstract

**Purpose:**

To investigate the choroideremia (*CHM*) gene in two families with CHM and to characterize the related clinical features.

**Methods:**

Two families underwent complete ophthalmic examinations and three males were diagnosed with CHM. Genomic DNA was extracted from the leukocytes of peripheral blood collected from the two families and from 100 unrelated control subjects from the same population. Exons 1–15 of *CHM* were amplified by PCR and directly sequenced. Ophthalmic examinations included best-corrected visual acuity, slit-lamp examination, fundus examination, visual field, optical coherence tomography, electroretinogram, and Pentacam.

**Results:**

The affected men were hemizygous and had night blindness, chorioretinal atrophy spreading from the posterior pole to the mid-periphery, and bareness of the sclera. A novel c.1488delGinsATAAC mutation was detected in *CHM* in family 1. Another mutation c.1703 C>G (S558X) within exon 14 of *CHM* was identified in family 2, which caused the serine 558 codon (TCA) to be changed to a stop codon (TGA).

**Conclusions:**

This study identified a novel mutation in *CHM* associated with CHM and its related clinical features. Our findings expand the genotypic spectrum of *CHM* mutations associated with CHM and confirm the role of Rab escort protein-1 in the pathogenesis of CHM.

## Introduction

Choroideremia (CHM) has been recognized as clinically distinct from other retinal degenerations for more than half a century, and is known to be a genetic heterogeneous X-linked recessive disease associated with different types of mutations in the CHM gene (*CHM*) [1]. Affected males develop night blindness in their teenage years, followed by loss of peripheral vision because of the progressive visual field constriction, and blindness. The incidence of CHM is estimated as 1 in 100,000 [1].

The *CHM* gene comprises 15 coding exons that span a genomic sequence of approximately 186 kb, which is made up of 653 amino acids and located on chromosome Xq21.2 [2]. It encodes Rab escort protein-1 (REP-1), which functions in the prenylation of small Rab proteins in the eye. REPs can bind to newly synthesized Rab protein to escort them to Rab GGTases [3,4]. They contain three sequence-conserved regions (SCR1, SCR2, and SCR3) that are similar in sequence to Rab GDP-dissociation inhibitor proteins [5,6]. Crystal structure analysis of REP proteins reveals two conserved domains: a multisheet domain I and a smaller globular α-helical domain II [5,6]. The N-terminal half of GDP-dissociation inhibitor proteins (the first 450 amino acids) shares conserved regions (SCRs 1–3) with members of the REP family. Some of the nonsense and frameshift mutations have been observed in the C-terminal part in CHM patients [5].

So far, more than 100 different pathogenic *CHM* mutations have been reported, most leading to a complete loss of the gene product REP-1 [7,8]. The spectrum of defects identified in *CHM* of patients with CHM includes translocations, deletions, and a variety of subtle mutations [9-17]. Deletions vary in extent from deletion of a few kilobases, to removal of several exons, to removal of the complete *CHM* gene and virtually the entire Xq21 band [7,10,16,18]. This study reports the mutational analysis of two CHM families at the gene level and related clinical features, and identifies two truncating mutations.

## Methods

**Figure 1:**
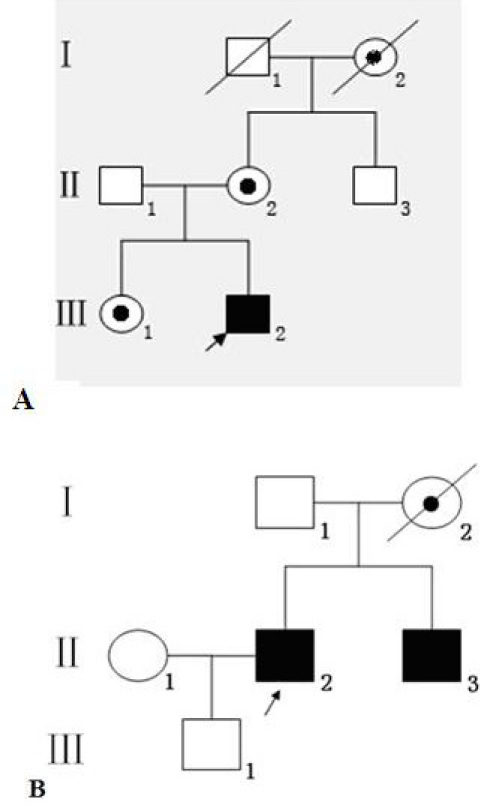
The pedigree of Chinese families with choroideremia. Square symbols denote males, and circular symbols denote females. The shaded symbols indicate ophthalmologist-confirmed choroideremia (CHM). The circles with a dot indicate female carriers. The arrow points to the proband. The transmission pattern suggested that the CHM was inherited in an X-linked hereditary manner. Panel **A** represents Family 1, panel **B** represents Family 2.

Two families were diagnosed as having CHM (Figure 1) at the Zhongshan Ophthalmic Center. We performed the ophthalmic examinations as follows: Visual acuity was examined using the ETDRS chart (Precision Vision, La Salle, IL). Fundus photograph and fundus fluorescein angiography imaging was performed using a Heidelberg Retina Angiograph (Heidelberg Engineering, Heidelberg, Germany). The NIHON KOHDEN electroretinogram (ERG) system (Neuropack, Tokyo, Japan) was used to assess the amplitudes of the rod and cone responses, and optical coherence tomography (OCT) scans (TOPCON, Tokyo, Japan) were used to assess the thickness and pathology of the posterior pole of the retina. Anterior segment measurements were measured with Pentacam HR version 70700 (Oculus, Weltzar, Germany). In addition, physical examinations were performed to exclude systemic diseases.

### Sample collection

The two affected families were identified at the Zhongshan Ophthalmic Center. One hundred subjects without diagnostic features of CHM from the same population were recruited to serve as normal controls. After informed consent was obtained from all participating individuals following the principles of the Declaration of Helsinki, venous blood samples were collected for genomic DNA extraction from peripheral blood leucocytes using standard protocols.

### Mutation detection

All coding exons and their flanking regions were amplified by PCR with the primers listed in Table 1 [11]. The PCR products were sequenced from both directions with the ABI3730 Automated Sequencer (PE Biosystems, Foster City, CA). The sequencing results were analyzed using Chromas (version 2.3) and compared with the reference sequences in the NCBI database.

**Table 1:** Primers used for PCR.

**Exon**	**Forward (5′-3′)**	**Reverse (5′-3′)**	**Product size (bp)**
1	GACCTTCCACCCAAGAACTAC	ACAGTCTTCCTAAACTTTGTCC	216
2	TGTTCTATACAGCAATGGCA	GGAAATATTAAATGCTATCGTT	190
3	TAAGGGTTAAAGATGGTTTGTTGATG	TTTCTTCAGTGCAGGGTTACTATGTA	236
4	TTGCATGTTTCACACTGCCCAC	AGTCATTAATTTAGTTTACCTGCAG	222
5.1	AGCCTGGTGTTTATTATTATATT	GTCACTTCAGCACCATTTAC	246
5.2	GATCCAGAGAATGCGCTAG	GCAAAGATGGGTAAAATTAGT	246
6	CCAATTTTTCTACTATTTCAAC	AACTTAAGCTGATGCCCAGT	214
7	AGTTATATCATTAGGAAGCAG	TTGGAGAGCACTACTTAATG	206
8	AGTTTAGTTCTGATTTTAAGTG	CACTTTTAGAAGGGACAAGAA	309
9	TTTTCAACCCAATTACCCTA	TATATATGAAGGTTACTTATATC	156
10	ATGAACTTTTATGGTATGCTTATCTT	GTCAATAAAATTACCTTCGCTTGC	185
11	CGAAACTTATCCATGGAATC	GTGTAGTGATTAGTTCACCA	207
12	GATCTAACAGCTGTGTCTGAT	AAAATACAAATAACCACTCT	174
13	GCTCAGCTCTCTATTATCCAT	GAAGATTATGATGGTTACAT	258
14	TAGGCTACACAGTGTAGTAA	GACTTCTCTCCTCCCAGAGG	322
15	AGTTAATGCCAGAAATGCAC	GGGTATCCAGTTTGGTGTATA	289

Summary of the primers and products length used for the amplification of the all exons of *CHM*.

## Results

### Clinical data

**Figure 2:**
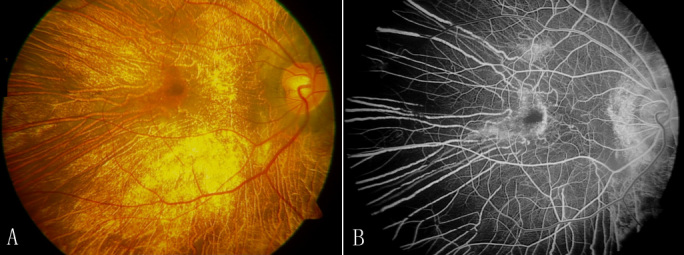
Fundus photographs of the right eye of the 38-year-old choroideremia-affected proband of family 1 show symmetric profound chorioretinal atrophy with preservation of the central macula. **A**: The fundus shows areas of retinal pigment epithelium (RPE) disruption, severe chorioretinal atrophy, loss of choriocapillaris, and bare sclera. **B**: Fluorescein angiography of the same affected male patient shows extensive chorioretinal atrophy with preservation of an island of RPE at the macular area.

The probands of family 1 and family 2 were diagnosed as having bilateral CHM. Physical examinations excluded systemic disorders in all patients. The clinical findings showed that the best-corrected visual acuity of the proband (III-2) of family 1 was 0.3 OD (LogMAR) and 0.4 OS with myopia of −2.25D OD and −1.75D OS. Unlike the normal homogeneous brown background of melanin pigment in the normal retinal pigment epithelium (RPE) and choroid, the retina of the proband showed symmetric profound chorioretinal atrophy with preservation of the central macula. A fundus photograph (Figure 2A) of the right eye of the 38-year-old CHM-affected proband of family 1 showed symmetric profound chorioretinal atrophy, areas of RPE disruption, loss of choriocapillaris, and bare sclera. Fluorescein angiography of the same affected male patient (Figure 2B) showed extensive chorioretinal atrophy with preservation of an island of RPE at the macular area.

**Figure 3:**
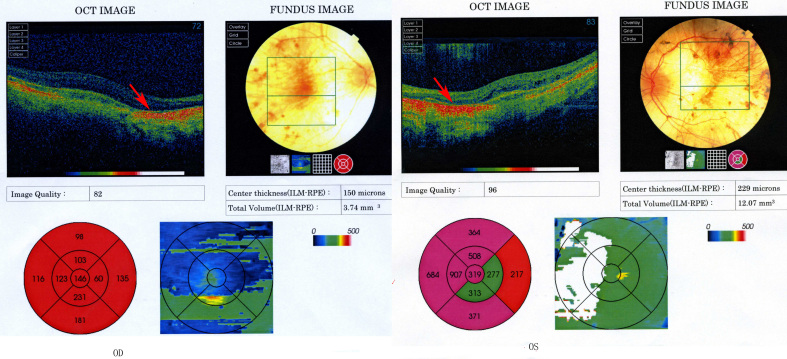
Optical coherence tomography shows the retina of the proband to be thinner than normal; the signal was stronger in the some parts of the retina because of the choriocapillaris atrophy and the bare sclera (arrow).

Visual field examination showed only a central 5 degrees of visual field (tunnel vision). OCT demonstrated that the retina of the proband was thinner than normal and the signal was stronger in some parts of the retina because of the choriocapillaris atrophy and the bare sclera (Figure 3). ERG showed no rod and cone responses according to the ERG standards of the International Society for Clinical Electrophysiology of Vision (ISCEV, 2008 Version) [19].

There were no significant differences in the anterior segment parameters between the affected patients and the controls (p>0.05). The examination findings of the affected men in family 2 were almost the same as those found in the family 1 proband.

The mother (II-2) of the proband in family 1 had poor visual acuity and visual field, whereas the older sister showed good visual function in terms of both visual acuity and visual field. Because the family lived some distance from Zhongshan Ophthalmic Center, we were unable to obtain blood samples from additional family members for mutation screening.

### Mutation screening

**Figure 4:**
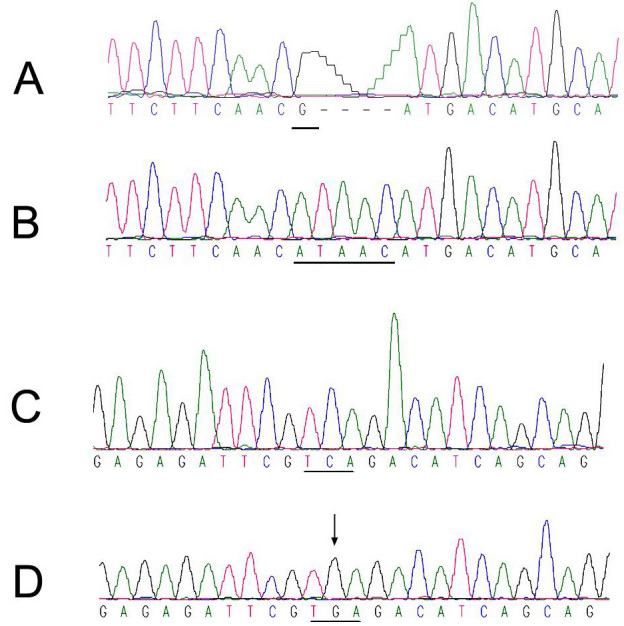
DNA sequence of a part of the *CHM* gene in the unaffected men and affected individuals. **A** represents normal sequence. **B** represents a hemizygous mutation 1488delGinsATTAC in the affected man. This mutation is predicted to truncate the 653 amino acid choroideremia (CHM) protein by 157 amino acids because of the insertion of the stop codon TAA. **C** represents normal sequence. **D** represents a C to G transversion at nucleotide 1703.

Compared to the unaffected patients (Figure 4A), sequencing of the complete coding region of *CHM* in the affected member of family 1 showed a hemizygous mutation c.1488delGinsATTAC (Figure 4B). This mutation is predicted to truncate the 653 amino acid CHM protein by 157 amino acids. Sequencing of the complete coding region of *CHM* in the affected member of family 2 showed a hemizygous mutation of a C to G transversion at nucleotide 1703, changing the serine 558 codon (TCA; Figure 4C) to a stop codon (TGA; Figure 4D). The mutation c.1703 C>G (S558X) created a new restriction site for BsmAI, permitting convenient DNA-based diagnosis among the family members. This alteration is expected to result in a product that would lack the last 96 amino acids, predicting a truncated and nonfunctional REP-1. These were the only changes seen in the affected men in the two families. The alterations were not seen in 100 unrelated control subjects (200 chromosomes) from the same population, tested by bidirectional sequence analysis. No other mutations were observed in the other exons.

## Discussion

In this study, we found two mutations in the two exons of *CHM* that are associated with CHM: c.1488delGinsATAAC and c.1703 C>G. These two mutations, rather than a rare polymorphism in the normal population, are the causative mutations in the two CHM families, respectively.

The novel c.1488delGinsATAAC mutation detected in the CHM family 1 created a new premature stop codon that indicates truncation of the C-terminal 157 residues of REP-1, leaving only 496 amino acids of the 653 residules. So far, nonsense, deletion, or insertion mutations have been detected in *CHM,* and most of these will result in truncation of the encoded protein [13]. However, indels in *CHM* have not been reported previously.

Our data demonstrating the truncation in the *CHM* gene from CHM patients suggest that a REP-1 protein lacking only 157 amino acids at the carboxy terminus is unable to function as a subunit of Rab GG transferase in vivo. It is likely that this abnormal REP-1 is degraded by enzymatically.

The mutation c.1703 C>G (S558X) within exon 14 of the *CHM* gene, which was found in CHM family 2, was previously described in one family in southern France [20]. Direct DNA sequencing revealed a C to G transversion at nucleotide 1703, changing the serine 558 codon (TCA) to a stop codon (TGA). Mutation c.1703 C>G (S558X) creates a new restriction site for BsmAI, permitting convenient DNA-based diagnosis in the family. This alteration is expected to result in a product that would lack the last 96 amino acids, predicting a truncated and nonfunctional REP-1. The c.1703 C>G (S558X) mutation occurring in one of the Chinese families in our study was also reported in a French family, which might indicate that the nucleotide 1703 is easily damaged.

In this study, we performed a detailed investigation of the clinical features of CHM. Affected males in the two families of this study all developed night blindness in their teenage years, followed by loss of peripheral vision due to progressive visual field constriction and blindness. Moreover, affected patients in both families all had various grades of myopia. Most previous studies did not provide the details of the refractive errors of the patients, but Binkhorst reported a patient with CHM who also had myopia [21]. We speculate that there is a close relationship between myopia and CHM.

The defect in the choroid and the fragility of the blood-retina barrier, as observed on OCT in our patients, make the eye vulnerable to various kinds of damage and other diseases. Krock and coauthors [22] have documented that mutations in Rep1 disrupt cellular processes in the RPE, and these cause photoreceptor death. Patients with CHM have been reported as having cataract [21], cystic macular edema [23], and recurrent uveitis [24].

CHM is a rare eye disease with clinical features similar to those of RP. So far, no effective treatment exists for either disease. Transplantation of autologous transduced iris pigment epithelial (IPE) cells into the subretinal space might help CHM patients in the future [25]. In animal studies, several neurotrophic factor genes have been transduced into IPE cells with adeno-associated virus, plasmid vector, or adenovirus, and the transplantation of those transduced IPE cells may help rescue photoreceptor cells from several types of photoreceptor damage in animals [25].

In summary, we identified two families with CHM of which the affected men were hemizygous and had night blindness, chorioretinal atrophy spreading from the posterior pole to the mid-periphery, and bareness of the sclera. Two truncating mutations were found in these two families, each with one distinct mutation. One was a novel mutation not previously reported: c.1488delGinsATAAC.
